# Genetics of type 2 diabetes mellitus in Indian and Global Population: A Review

**DOI:** 10.1186/s43042-022-00346-1

**Published:** 2022-09-02

**Authors:** Anjaly Joseph, Maradana Thirupathamma, Elezebeth Mathews, Manickavelu Alagu

**Affiliations:** 1 Department of Public Health and Community Medicine, Central University of Kerala, Kasaragod, Kerala 671320 India; 2 Department of Genomic Science, Central University of Kerala, Kasaragod, Kerala 671320 India

**Keywords:** Type 2 diabetes, Genetics, Marker–trait association, Risk alleles, SNP

## Abstract

**Background:**

Non-communicable diseases such as cardiovascular diseases, respiratory diseases and diabetes contribute to the majority of deaths in India. Public health programmes on non-communicable diseases (NCD) prevention primarily target the behavioural risk factors of the population. Hereditary is known as a risk factor for most NCDs, specifically, type 2 diabetes mellitus (T2DM), and hence, understanding of the genetic markers of T2DM may facilitate prevention, early case detection and management.

**Main body:**

We reviewed the studies that explored marker–trait association with type 2 diabetes mellitus globally, with emphasis on India. Globally, single nucleotide polymorphisms (SNPs) rs7903146 of Transcription Factor 7-like 2 (TCF7L2) gene was common, though there were alleles that were unique to specific populations. Within India, the state-wise data were also taken to foresee the distribution of risk/susceptible alleles. The findings from India showcased the common and unique alleles for each region.

**Conclusion:**

Exploring the known and unknown genetic determinants might assist in risk prediction before the onset of behavioural risk factors and deploy prevention measures. Most studies were conducted in non-representative groups with inherent limitations such as smaller sample size or looking into only specific marker–trait associations. Genome-wide association studies using data from extensive prospective studies are required in highly prevalent regions worldwide. Further research is required to understand the singular effect and the interaction of genes in predicting diabetes mellitus and other comorbidities.

## Background

According to the International Diabetes Federation (IDF), “Diabetes Mellitus is one of the fastest growing global health emergencies of the twenty-first century” [1]. The global prevalence of type 2 diabetes mellitus (T2DM) was 536.6 million in 2021 and is projected to increase to 642.7 million by 2030 and 783.2 million by 2045, which is almost 46% increase in the prevalence [1]. It is estimated that the highest percentage increase will be in middle-income countries compared to high- and low-income countries [1]. The highest prevalence of diabetes in people aged 20–79 years is reported in the Middle East and North African region (MENA) (18.1%). In contrast, the African region has the lowest prevalence (5.2%) which was attributed to comparatively low levels of urbanization and low levels of obesity [1]. China (140.9 million), India (74.2 million) and Pakistan (33 million) have the most significant number of adults with T2DM and are expected to remain the same in 2045. Almost one in two adults with diabetes is undiagnosed, and 87.5% of the undiagnosed are in middle- and low-income countries. In 2021, excluding the mortality associated with the COVID-19 pandemic, almost 6.7 million between the age of 20–79 years died due to diabetes-related complications which is almost 12.2% of the global deaths from all causes [1]. Among this, almost 32.6% of the deaths occurred in working-age people [1]. Diabetes-related costs have increased by 316% over the past 15 years. This highlights the urgent need to improve the ability to prevent the development of T2DM at an early stage.

The increasing burden of T2DM is not completely understood as the aetiology of diabetes is multifactorial, including genetic factors coupled with environmental factors such as rapid urbanization, urban migration, and lifestyle changes [2]. Decades of rapid urbanization and associated socio-economic transformation have resulted in healthier lifestyles and dietary preferences shifting to unhealthy practises [3]. Environmental factors play a significant role in the development of diabetes, but they do not impact everyone in the same way. Even with the same environmental exposures, some are more susceptible to developing diabetes than others, and this increased risk is considered to be inherited [4].

Currently, there are non-clinical and clinical measures for diabetes prevention and management such as lifestyle modifications (dietary modifications, physical activity, behavioural modifications), medical nutrition therapy (MNT), bariatric surgeries, treatment using medicinal plants [5, 6] and clinical measures such as oral anti-diabetic drugs and insulin [7]. Although the benefits of lifestyle modification in diabetes prevention and the effectiveness of pharmacological treatments are well approved, there continues the increase in the prevalence of T2DM. Research suggests that the T2DM has a critical genetic predisposition. Evidence indicates that Indians are more susceptible to insulin resistance than Europeans of similar age and body mass index, suggesting the significant possibility of population-specific genetic risk factors [4, 8, 9]. Furthermore, studies have identified that South Asians have a greater tendency for visceral fat deposition, higher total body fat percentage and insulin resistance compared to other ethnic groups at similar levels of body mass index [4]. Epidemiological studies have reported that migrant Asian Indians living in different parts of the world show a much higher prevalence of diabetes than the residents of countries [4].

Genetic factors play an important role in the pathogenesis of diabetes and thus are an essential element in understanding the cause of the disease and possible prevention methods. Advances in genotyping and sequencing have led to the identification of SNP as genetic variants associated with type 2 diabetes or related glycaemic traits [9]. Combined genetic risk scores composed of the weighted sum of the risk alleles at these loci have been tested for their ability to predict diabetes in individuals beyond the information provided by clinical risk factors [10]. Genome-wide association mapping is a concept well utilized for identifying new risk alleles/loci. Developed countries like the USA and Nordic countries have initiated the Precision Medicine Initiative (PMI) for some major non-communicable diseases such as cancer and T2DM [11]. The emerging field of precision medicine requires understanding the risk alleles of each population. This review aims to analyse the known genetic factors of T2DM in the global population, including India, and identify the significant risk alleles.

## Materials and methods

In this review, we included original research and meta-analysis studies that assessed the genetic determinants of T2DM among people in the Indian subcontinent and globally, published to date (the year 2021). We included studies that explored marker–trait associations from observational studies (*n* = 59) and genome-wide association studies (GWAS) (*n* = 6). Those studies that reported the criteria of diagnosis for type 2 diabetes mellitus using World Health Organization (WHO) [12] or American Diabetes Association (ADA) criteria [13] only were included in the review. Genome-wide association study reports are from peer-reviewed published works where the samples were taken based on the standard procedure and traits.

### Search strategy and study selection

**Fig. 1:**
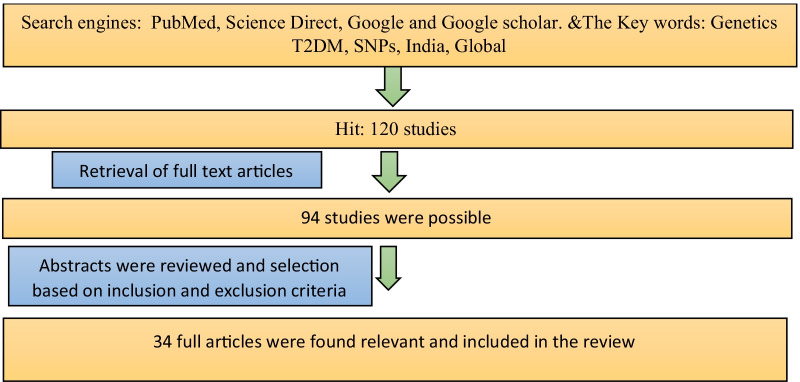
Flowchart of data extraction

Data sources such as PubMed, Google and Google Scholar were used to identify the studies. The keywords used were “Genetics of T2DM”, “SNPs”, “India” and “Global”. The flowchart of data extraction and review is given in Fig. 1. We classified the SNPs associated with T2DM according to regions such as South East Asian region (India, Sri Lanka), Western Pacific region (Japan, Australia), Eastern Mediterranean (Jordan), African region (Western Africa, Ghana, Nigeria and Kenya) and European countries and American regions (Mexico, Latin America, the USA).

### Genetics of T2DM

Type 2 diabetes mellitus is polygenic, and over 100 genes have already been reported [4]. Three primary methods are adopted to identify the genetic predisposition of T2DM, which primarily focuses on linkage peaks from family studies, candidate genes on a biological basis and genome-wide association analysis.

Family and twin studies have indicated 20–80% of inheritability of diabetes [14]. First-degree relatives of individuals with T2DM were three times more likely to develop the disease than individuals without a positive family history [14]. Studies have reported that individuals born to affected parents were more likely to develop T2DM [odds ratio (OR) = 6.1, 95% CI = 2.9–13] compared to people with unaffected parents (OR = 3.4–3.5). Although maternal and paternal diabetes conferred risk for developing diabetes, the Framingham offspring study reported that offspring with maternal diabetes had a slightly more chance for abnormal glucose tolerance than those with paternal diabetes (OR = 1.6, CI = 1.1–2.4) [1]. Multiple twin concordance studies in T2DM reported a higher concordance rate in monozygotic twins (OR: 0.29–1.00) than in dizygotic twins (OR: 0.10–0.43), indicating a significant genetic component of the disease [14].

A candidate gene is a gene whose chromosomal location is associated with a trait of interest. Because of its location, the gene is suspected of causing the disease or other related phenotype [15]. Candidate gene association studies focussed on the association of pre-specified genes of interest and the disease. The genes that were found to be associated with T2DM include peroxisome proliferator-activated receptor gamma (*PPARG*), insulin receptor substrate 1 (*IRS1*) and *IRS-2*, potassium inwardly rectifying channel, subfamily J, member 11 (*KCNJ11*), Wolfram syndrome 1 (wolframin) (*WFS1*), hepatocyte nuclear factor-1 alpha (*HNF1A*), HNF1 homeobox B (*HNF1B*) and *HNF4A* [14]. The genes, including Rap guanine nucleotide exchange factor 1 (*RAPGEF1)* and tumour protein 53 (*TP53)* were identified using an algorithm that prioritized candidate genes for complex human traits based on trait-relevant functional annotation but had not been consistently replicated in later studies [4]. Other candidate genes are tyrosine-protein kinase (*LYN),* DENN domain-containing protein 1B (*DENND1B),* mitochondrial ribosomal protein *(MRPL30)* 3-hydroxyisobutyrate dehydrogenase (*HIBADH)* [16]. *PPARG* and *KCNJ* [11] were the most validated diabetes-associated genes identified through functional candidate analysis [17]. With the rapid improvements in the genotyping technology of SNPs and the Hap Map project, the methods for identifying susceptibility genes have changed dramatically [18]. GWAS identified more than 70 genetic variants associated with T2DM [4]. These gene variants were related to different metabolic pathways of the disease. Studies conducted among European communities have identified 41 SNPs associated with T2DM and found that genes associated with glucose homeostasis, insulin pathway and pancreatic development pathways were the candidate genes associated with T2DM [19]. SNPs at high mobility group box 1 pseudogene 1 (*HMG1L1)*/CCCTC-binding-like factor (*CTCFL)*, paired box 4 A4 (*PLXNA4)*, cleavage-activating protein (*SCAP)*, chr5p11 and a novel locus at 13q12 at sarcoglycan gamma (SGCG) were associated with T2DM [20]. Table 1 provides a comprehensive list of marker–trait association of T2DM, and includes significant findings related to the genes of T2DM in the global population, including India. Table 2 describes the functional classification of major genes related to T2DM and their related morbidity.

**Table 1:** Comprehensive list of marker–trait association of T2DM: the studies covering the global population, including India are taken and the significant findings related to the genes of T2DM are listed here

Nos.	Gene	SNP (P/R)	Risk allele/non-risk allele	OR (95% CI), P value	Population	Type of study
1	Peroxisome proliferator-activated receptor gamma (PPARG)	1. rs1801282 [4, 24, 53] (P)	C/G [53]	India—1.37 (1.19–1.59), 1.6 × 10^−5^ [53] Europeans—1.14 (1.08–1.20), 1.7 * 10^–6^ [53]	India (Delhi, Pune); Europeans [53]	Observational case–control study [53]
				0.7 (0.50–0.99), 0.046 [24]	Hyderabad [24]	Observational case–control study [24]
			CC + CG versus GG [4]	0.13 (0.03–0.56), 0.0007 [4]	Sikh Indians (Punjab, Haryana, Himachal Pradesh, Delhi and Jammu and Kashmir [53])	Observational case–control study [53]
		2. rs11715073 [4] (P)	CC + CG versus GG [4]	0.52 (0.31–0.86), 0. .010 [4]	Sikh Indians (Punjab, Haryana, Himachal Pradesh, Delhi and Jammu and Kashmir [4])	Observational case–control study [4]
		3. rs2972164 [4] (R)	CC versus CT + TT	1.30 (1.00–1.68), 0. .048 [4]	Sikh Indians (Punjab, Haryana, Himachal Pradesh, Delhi and Jammu and Kashmir [4])	Observational case–control study [4]
2	Potassium Inwardly Rectifying Channel Subfamily J Member 11 (KCNJ11)	1. rs5219 [27, 53] (R)	T/C	India—1.39 (1.26–1.54), 6.7 * 10^–11^ [53] Europeans—1.14 (1.10–1.19), 6.7 * 10^–11^ [53]	India (Delhi, Pune); Europeans [53]	Observational case–control study [53]
				1.14 (1.10–1.1), 5 * 10^–11^ [27]	UK [27]	Observational case–control study [27]
		2. rs5215 [21] (P)	CC	0.42 (0.28–0.62) *P*-0.0001[21]	South Indian [21]	Observational case–control study [21]
3	Transcription factor 7-like 2 (TCF7L2)	1. rs7903146 [16, 18, 24, 53–55] (R)	T/C	India—1.89 (1.71–2.09), 4.6 * 10^–34^ [53] Europeans—1.37 (1.31–1.43),1*10^–48^ [53]	India (Delhi, Pune); Europeans [53]	Observational case–control study [53]
				1.32 (1.22–1.43),5.3 * 10^–13^ [16]	Africa [16]	GWAS [16]
				1.44 (1.17–1.78),2.05^–08^ [54]	India—Punjab, Jammu and Kashmir, Orissa [54]	Observational case–control study [54]
				1.35 (1.19–1.54), 6.1 × 10^−6^ [18]	Sri Lanka [18]	Observational case–control study [18]
				1.62 (1.17–2.25), 0.003 [55]	Egypt [55]	Observational case–control study [55]
			CT	1.99 (1.49–2.64), < 0.001 [24]	Hyderabad [24]	Observational case–control study [24]
			TT	3.58 (2.09–6.13), < 0.001 [24]	Hyderabad [24]	Observational case–control study [24]
		2. rs12255372 [54–56] (R)	G/T	2.06 (1.45–2.93), < 0.0001 [55]	Egypt [55]	Observational case–control study [55]
				2.14 (1.01–4.55),0.004 [56]	Mexico [56]	Observational study [56]
				1.3376, P value-1.46E-06 [54]	India—Punjab, Jammu and Kashmir, Orissa [54]	Observational case–control study [54]
			GG	Chi-square-30.73 < .0001[56]	Mexico [56]	Observational study [56]
4	Solute carrier family 30, member 8 *(*SLC30A8)	1. rs13266634 [18, 19, 53] (R)	C/T	Indians—1.34 (1.20–1.50), 3.4 * 10^–7^ Europeans—1.12 (1.07–1.16), 5.3 * 10^–8^ [53]	India (Delhi, Pune); Europeans [53]	Observational case–control study
				1.33 (1.14–1.55), 3.0 × 10^−4^ [18]	Sri Lanka [18]	Observational case–control study [18]
				3.24 (1.20–13.39) [19]	Germany [19]	Observational case–control study [19]
5	Haematopoietically expressed homeobox protein (HHEX)	1. rs1111875 [53, 54, 57, 57] (R)	G/A	Indians—1.27 (1.16–1.39), 5.7 * 10^–7^ Europeans—1.13 (1.09–1.17), 5.7 * 10^–10^ [53]	India (Delhi, Pune); Europeans [53]	Observational case–control study [53]
				120.82 (17–inf*), < 0.001 [57]	Iran [57]	Observational case–control study [57]
				1.262, 1.44^–05^ [54]	India—Punjab, Jammu and Kashmir, Orissa [54]	Observational case–control study [54]
			GG	32.32 (13–91), < 0.001 [57]	Iran [57]	Observational case–control study [57]
		2. rs5015480 [54, 58] (R)	C/T	Chi-square-19.94, 1.55^–03^ [58]	Korea [58]	GWAS [58]
				1.2943, 1.83^–06^ [54]	India—Punjab, Jammu and Kashmir, Orissa [54]	Observational case–control study [54]
6	Cyclin-dependent kinase inhibitor 2A (CDKN2A)	1. rs10811661 [53] (R)	T/C	Indians—1.37 (1.18–1.59), 5.1 * 10^–5^ [53] Europeans—1.20 (1.14–1.25),7.8 * 10^–25^ [53]	India (Delhi, Pune); Europeans [53]	Observational case–control study [53]
7	Insulin-like growth factor 2 mRNA-binding protein 2 (IGF2BP2)	1. rs4402960 [53] (R)	T/G	Indians—1.20 (1.09–1.33), 2.6 * 10^–3^ Europeans—1.14 (1.11–1.18), 8.9 * 10^–36^ [52]	India (Delhi, Pune); Europeans [53]	Observational case–control study [53]
8	Cdk5 regulatory associated protein 1-like 1 (CDKAL1)	1. rs10946398 [53] (R)	C/A	Indians—1.18 (1.07–1.32), 1.6 * 10^–3^ Europeans—1.12 (1.08–1.16), 4.1 * 10^–11^ [53]	India (Delhi, Pune); Europeans [53]	Observational case–control study [53]
		2. rs7756992 [24]	GG	1.97 (1.09–3.56), 0.025 [24]	Hyderabad [24]	Observational case–control study [24]
		3. rs7754840 [59] (R)	GG	1.36 (1.02–1.82), 0.0349 [59]	Han Chinese [59]	Observational case–control study [59]
			CC	2.03 (1.42–2.89), 9.07 * 10^–5^ [59]	Han Chinese [59]	Observational case–control study [59]
		4. rs4712523 [59] (R)	AG	1.38 (1.03–1.84), 0.0291[59]	Han Chinese [59]	Observational case–control study [59]
			GG	1.97 (1.39–2.81) 1.49 × 10^–4^ [59]	Han Chinese [59]	Observational case–control study [59]
		5. rs4712524 [59] (R)	AA	1.42 (1.06–1.90),0.0189 [59]	Han Chinese [59]	Observational case–control study [59]
			GG	1.85 (1.30–2.62) 5.85 * 10^–4^ [59]	Han Chinese [59]	Observational case–control study [59]
9	Alkylglycerol Monooxygenase (AGMO)	1. rs73284431 [16] (R)	G /C	1.48 (1.30–1.69), 5.2 * 10^–9^ [16]	Africa [16]	Observational case–control study [16]
10	Adiponectin, C1Q And Collagen Domain Containing (ADIPOQ)	1. rs182052 [53] (R)	GG	1.23 (1.02–1.48), 0.027 [53]	Sikh Indians (Punjab, Haryana, Himachal Pradesh, Delhi and Jammu and Kashmir [53])	Observational case–control study [53]
		2. rs7649121 [53] (R)	AA	1.36 (1.03–1.79), 0.029 [53]	Sikh Indians (Punjab, Haryana, Himachal Pradesh, Delhi and Jammu and Kashmir [53])	Observational case–control study [53]
		3. rs1501299 [47] (R)	GG [47]	2.350 (1.231–4.486), 0.010 [47]	Jordan [47]	Observational study [47]
			TT [47]	4.774 (1.551–14.693) 0.006 [47]	Jordan [47]	Observational study [p47]
11	Insulin-Degrading Enzyme (IDE)	1. rs1887922 [54] (R)	T/C [54]	1.3423, 5.44^–05^	India—Punjab, Jammu and Kashmir, Orissa [54]	Observational case–control study [54]
12	Ectonucleotide pyrophosphatase/phosphodiesterase 1 (ENPP1)	1. rs1044498 [54] (R)	A/C [54]	1.341,4.24–^05^ [55]	India—Punjab, Jammu and Kashmir, Orissa [54]	Observational case–control study [54]
13	Alpha-Ketoglutarate-Dependent Dioxygenase (FTO)	1. rs9939609 [54] (R)	T/A [54]	1.2906, 7.38^–06^ [54]	India—Punjab, Jammu and Kashmir, Orissa [54]	Observational case–control study [54]
		2. rs9940128 [26] (R)	AG	1.48 (1.16–1.89), 0.001 [26]	Chennai [26]	Observational case–control study [26]
			GG	2.04 (1.42–2.94), < 0.0001[26]	Chennai [26]	Observational case–control study [26]
		3. rs1588413 [26] (R)	CT	1.81 (1.43–2.31), < 0.0001[26]	Chennai [26]	Observational case–control study [26]
			TT	1.86 (1.18–2.92), 0.007 [26]	Chennai [26]	Observational case–control study [26]
		4. rs11076023 [26] (P)	AT	0.70 (0.53–0.92), 0.01 [26]	Chennai [26]	Observational case–control study [26]
			TT	0.64 (0.46–0.89), 0.008 [26]	Chennai [26]	Observational case–control study [26]
14	Cyclin-Dependent Kinase Inhibitor 2B CDKN2B	1. rs564398 [27] (R)	C/T	1.12,1.2 × 10^−7^ [27]	UK population [27]	Observational case–control study [27]
15	Solute Carrier Family 16 Member 11 (SLC16A11)	1. rs77086571[27] (R)	C/T	1.29,5.4 × 10^−12^[27]	UK population [27]	Observational case–control study [27]
16	Dual specificity phosphatase 9 (DUSP9)	1. rs5945326 [19] (R)		2.04 (1.14–3.89) [19]	European population [19]	Observational study [19]
17	Kruppel-like factor 14 (KLF14)	1. rs972283 [19] (R)		3.45 (1.69–8.38) [19]	European population [19]	Observational study [19]
18	Ribosomal protein S6 kinase alpha-1 (RPS6KA1)	1. rs1002487 [48] (R)	C	2.89^–11^ [48]	Arab [48]	GWAS [48]
19	Calcium-dependent secretion activator (CADPS)	1. rs487321 [48] (R)	A	2.054^–12^ [48]	Arab [48]	GWAS [48]
20	Valyl-tRNA synthetase (VARS)	1. rs707927 [48] (R)	G	1.61^–09^ [48]	Arab [48]	GWAS [48]
21	DExH-Box Helicase 58 (DHX 58)	1. rs12600570 (R) [48] (P)	T	1.48^–03^ [48]	Arab [48]	GWAS [48]
22	Retinoic acid-inducible gene (STRA6)	1. rs974456 [17] (P)	C/T	0.79 (0.69–0.91), 0.003 [17]	Kerala [17]	Observational case–control study [69]
		1. rs736118 [17] (P)	G/A	0.81 (0.71–0.93), 0.01 [17]	Kerala [17]	Observational case–control study [69]
		1. rs4886578 [17] (P)	G/A	0.74 (0.62–0.89), 0.0009 [17]	Kerala [17]	Observational case–control study [17]
23	Forkhead box protein A2 (FOXA2)	Polymorphism [60] (R)	A	1.44 (1.22–1.70), 1.5 9 10^–5^ [60]	North Indian [60]	Observational cohort study [60]

OR, odds ratio; CI, confidence interval; and P/R, protective/risk

**Table 2:** Functional classification of major genes related to T2DM and their related morbidity

Nos.	Gene	Major function	Disease	Population
1	Peroxisome proliferator-activated receptor gamma (PPARG)	Insulin resistance [53]	Central Obesity [53] Coronary heart disease [62] Acute Coronary Syndrome [61] Bladder cancer [62]	India (Delhi, Pune, Hyderabad, Punjab, Haryana, Himachal Pradesh, Delhi and Jammu and Kashmir, South Indians) [4, 24, 53] European region (Europeans, Sweden, Finland) [28, 29, 53] African region (South Africa) [40]
2	Potassium Inwardly Rectifying Channel Subfamily J Member 11 (KCNJ11)	Pancreatic beta-cell function [28]	Essential hypertension [63] familial persistent hyperinsulinemic hypoglycaemia of infancy (PHHI) [64, 65]	India (Delhi, Pune, South Indians) [21, 52] Europeans [28, 53] UK [23]
3	Transcription factor 7-like 2 (TCF7L2)	Regulating glucose tolerance [28]	IGT; T2DM [55] Cardiovascular diseases [65] Type 1 diabetes mellitus [66] Gestational diabetes mellitus [66] Latent autoimmune diabetes (LADA) [66] Obesity [66] Metabolic syndrome [66] Small bowel Crohn’s disease [66] Cancer (gastric, colon, rectal, lung, breast, prostate, renal) [66] Schizophrenia [66] Bipolar disorder [66] Cystic fibrosis [66] Premature adrenarche [66] Polycystic ovarian syndrome [66]	South East Asian region–India (Delhi, Pune, Punjab, Jammu and Kashmir, Orissa, Hyderabad [21, 53, 54]) South East Asian region (Sri Lanka) [18] Europeans [53] African region (South Africa) [16] Eastern Mediterranean (Egypt) [55] American region (Mexico) [56] UK [27]
4	Solute carrier family 30, member 8 *(*SLC30A8)	Insulin packaging and secretion [53]	Breast cancer [67] Glioblastoma tumours [67]	India (Delhi, Pune); Europeans [53] Germany [19] South East Asian region (Sri Lanka) [18]
5	Haematopoietically expressed homeobox protein (HHEX)	Maintains beta-cell differentiation and islet function [68]	Breast cancer [69]	India (Delhi, Pune, Punjab, Jammu and Kashmir, Orissa) [53, 55] Europeans [53, 58] Eastern Mediterranean (Iran [59]) Western Pacific region—Korea [57]
6	Cyclin-dependent kinase inhibitor 2A (CDKN2A)	Beta-cell function and regeneration [70]	Melanoma [71] Pancreatic cancer [71]	India (Delhi, Pune) [53] Europeans [53]
7	Insulin-like growth factor 2 mRNA-binding protein 2 (IGF2BP2)	Regulates cellular metabolism [72]	Obesity [72] Fatty liver [72] Cancer [72]	India (Delhi, Pune); Europeans [53]
8	Cdk5 regulatory associated protein 1-like 1 (CDKAL1)	Regulates mitochondrial activity and function in adipose tissue [73]	Pulmonary artery hypertension [74]	India (Delhi, Pune, Hyderabad) [24, 53] Europeans [53] China [59]
9	Alkylglycerol Monooxygenase (AGMO)	Catalysing the breakdown of alkylglycerols and lyso-alkyl glycerophospholipids [75]	Adolescent idiopathic scoliosis [75] Cancer [75] Tuberculosis [75] Autism spectrum disorders [75] Microcephaly [75] Neurodevelopmental disorders [75]	African region (South Africa) [16]
10	Adiponectin, C1Q And Collagen Domain Containing (ADIPOQ)	increases insulin sensitivity, enhances fatty acid oxidation, glucose uptake in skeletal muscles, reduces glucose uptake in liver [47]	prediabetes [47] Hypertension [76]	Sikh Indians (Punjab, Haryana, Himachal Pradesh, Delhi and Jammu and Kashmir [4]) Eastern Mediterranean (Jordan [47])
11	Insulin-Degrading Enzyme (IDE)	Insulin-degrading enzyme [77]	Alzheimer's diseases [77]	India—Punjab, Jammu and Kashmir, Orissa [54]
12	Ectonucleotide Pyrophosphatase/Phosphodiesterase 1 (ENPP1)	Negative regulator of insulin activity [28]	Generalized arterial calcification of infancy [78] Hypophosphatemic rickets [78]	India (Punjab, Orissa, Jammu and Kashmir) [54] Europeans [28]
13	Alpha-Ketoglutarate-Dependent Dioxygenase (FTO)	encodes for a non-heme and 2-oxoglutarate-dependent oxygenase with nucleic acid demethylase activity [58]	Obesity [26] Obesity related Hypertension [79] Colon cancer [80]	India (Punjab, Orissa, Jammu and Kashmir, Chennai) [26, 54]
14	Cyclin-Dependent Kinase Inhibitor 2B CDKN2B	Regulation of beta-cell mass, proliferation and insulin secretory function [81]	Glaucoma [82] Coronary artery disease [83]	UK population [27]
15	Solute Carrier Family 16 Member 11 (SLC16A11)	Hepatic liver metabolism [84]	Type 2 diabetes [84]	UK population [26]
16	Dual specificity phosphatase 9 (DUSP9)	Susceptibility to insulin resistance [85]	Prediabetes [19] Gestational diabetes mellitus [85]	European population [19]
17	Kruppel-like factor 14 (KLF14)	Improve insulin sensitivity and increase glucose uptake in Hepa1-6 cells [86]	Prediabetes [19] Metabolic syndrome [86]	European population [19]
18	Ribosomal protein S6 kinase alpha-1 (RPS6KA1)	Endothelial dysfunction [48]	Fasting plasma glucose [48]	Eastern Mediterranean (Arab [48])
19	Calcium-dependent secretion activator (CADPS)	Neural/endocrine-specific cytosolic and peripheral membrane protein required for the Ca2+ -regulated exocytosis of secretory vesicles [87]	Bipolar disorder Jejunal Somatostatinoma and Pineoblastoma [87]	Eastern Mediterranean (Arab [48])
20	Valyl-tRNA synthetase (VARS)	Linking amino acids [88]	Fasting plasma glucose [48] Neurodevelopmental disorders with microcephaly [88] Seizures [88] Cortical atrophy [88]	Eastern Mediterranean (Arab [48])
21	DExH-Box Helicase 58 (DHX 58)	Enables double-stranded RNA-binding [48]	–	Eastern Mediterranean (Arab [48])
22	Retinoic acid-inducible gene (STRA6)	Mediate cellular uptake of Vitamin A [89]	Gestational diabetes mellitus [89]	India (Kerala [17])
23	Forkhead box protein A2 (FOXA2)	Insulin resistance [90]	Parkinsonism [90]	India (North Indian [60])

### Genetic studies in the Indian population

South Asians have higher rates of T2DM compared to other ethnic populations. Migrant studies have also reported the same [17]. The most investigated functional candidate genes in South Asians include *PPARG*, *TCF7L2,* insulin-like growth factor 2 MRNA-binding protein 2 *(IG2BP2),* adiponectin, C1Q and collagen domain containing (*ADIPOQ)* and alpha-ketoglutarate-dependent dioxygenase (FTO) [4]. A study in Sri Lanka replicated the 36 SNPs associated with Europeans. Out of the 36 SNPs, 31 were significantly associated with T2DM. The strongest effects were seen at *TCF7L2* and solute carrier family 30, member 8 (*SLC30A8)* [18]. The Ala gene of PPARG was found to lower the 2-h plasma glucose among the Caucasians, while no effect was seen among the populations in Chennai. Sanghera et al. identified this gene's protective effect among the Sikh community of India [4]. A study conducted in seven geographically distinct areas of India explored 91 SNPs of 55 candidate genes and identified five genes associated with T2DM such as *TCF7L2* (rs7903146, rs12255372), insulin-degrading enzyme (*IDE)* (rs1887922), haematopoietically expressed homeobox protein *(HHEX)* (rs1111875, rs5015480), ectonucleotide pyrophosphatase/phosphodiesterase 1 *ENPP1* (rs1044498) and *FTO* (rs9939609, rs3751812) [12]. These genes play a major role in the metabolic pathways of diabetes pathobiology. The study also identified an increased risk (OR = 2.44, 95% CI = 1.67–3.59) when TCF7L2, HHEX, ENPP1 and FTO were combined [14]. KCNJ11 rs5210 and potassium voltage-gated channel subfamily Q member 1 (KCNQ1) rs2237895 variants were found to be significantly associated with risk of T2DM in the Indian population but were found insignificant in the South Indian population [21, 22].

A protective-odds (OR = 0.28, 95% CI = 0.19–0.43) was identified with a genotypic combination of IDE, HHEX, ENPP1 and FTO among controls. A study conducted among the Indo-European individuals in Delhi and Pune identified strong association at rs7903146 of *TCF7L2* with OR 1.67 [16]. A study by Radha et al. identified the association of rs4810424 and rs736823 of *HNF1A* gene with T2DM. Genome-wide studies have mapped a susceptibility locus for T2DM to 3q27, where ADIPOQ gene is situated. SNPs of this gene have been studied, and two SNPs, a silent T to G substitution in exon 2 and a G to T substitution in intron 2, were found to be associated in the Japanese population [20]. A study identified that + 10211T/G polymorphism in the adiponectin gene was associated with T2DM in the Asian Indian population [23]. In Hyderabad, South India, a study mapped 3 SNPs associated with T2DM rs7903146, rs12255372 and rs11196205. Among them, rs7903146 was more at risk for T2DM [24]. Initial European studies on the FTO gene identified rs9939609 as associated with high body mass index (BMI). In contrast, among South Indians, rs9939609 was associated with T2DM independent of body mass index (BMI). *TCF7L2* is the most widely studied gene, which has been positively associated with T2DM in Europeans [25]. The Chennai Urban Rural study showed similar results where rs12255372 and rs7903146 were associated with T2DM. The “T” allele of these SNPs showed association with non-obese participants. The variants rs9939609 T/A and rs7193144 C/T of FTO were associated with obesity in Asian Indians [26]. Recently, six variants—rs9940128, rs7193144, rs8050136 (intron 1), rs918031, rs1588413 (intron 8) and rs11076023 (3´UTR (unique transaction reference number)), across three regulatory regions of the *FTO* gene with obesity and T2D in a South Indian population showed that the rs9940128 A/G, rs1588413 C/T and rs11076023 A/T variants were associated with T2D but not with obesity [26]. The C/A variant of rs8050136 was associated with T2DM mediated through obesity. The haplotype “ACCTCT” of this SNP conferred a lower risk of T2DM in the South Indian population [26].

**Fig. 2:**
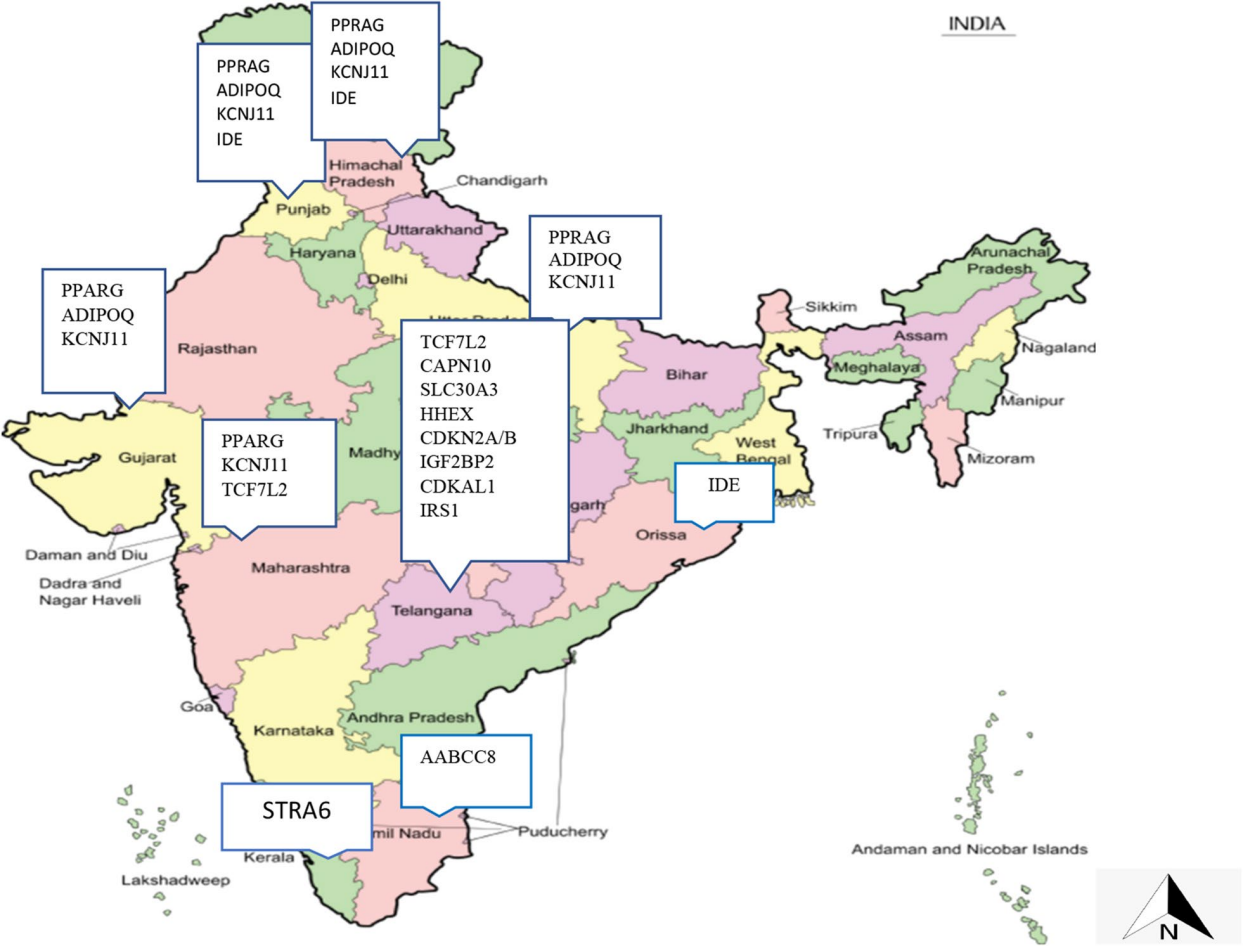
Mapping of SNPs associated with T2DM in India

A study in Kerala assessed SNPs of Retinoic acid-inducible gene (*STRA6*) (rs974456, rs351224, rs736118 and rs4886578), retinol-binding protein 4 (*RBP4)* (rs3758538, rs36014035 and rs34571439) and glucose transporter type 4 (*GLUT4)* (rs5412, rs5418 and rs5435). The SNPs of *STRA6* were associated with T2DM, while no association was found in *RBP4* and *GLUT4* 17]. Figure 2 shows the SNPs identified across the states of India.

### Genetic distribution of T2DM across regions of the world

Genetic studies in diverse populations are essential for several reasons. Identifying a population-specific variant associated with T2DM can help identify subjects at high risk in that population who could be selected for lifestyle or therapeutic, preventive intervention. Further, discovering causal genes in these populations can expand our understanding of T2DM or lead to a potential therapeutic target that could be valuable even in populations where the genetic variant that prompted the discovery is not present [14].

#### European region

The initial studies on the genes associated with T2DM were conducted among Europeans [27]. A study by Barroso et al. analysed 71 candidate genes based on their known or putative role in glucose metabolism. The selected genes were subdivided into three broad groups based on their function such as (1) genes primarily involved in pancreatic β-cell function; (2) genes primarily influencing insulin action and glucose metabolism in the main target tissues, muscle, liver and fat; and (3) other genes [28]. Twenty SNPs in 11 different genes showed statistically significant association with disease status (*p* < 0.05). The strongest statistical evidence for disease association was for genes such as son of sevenless homologue 1 (*SOS1),* phosphoinositide-3-kinase regulatory subunit 1 (*PIK3R1),* ATP-binding cassette subfamily C member 8 (*ABCC8),* insulin receptor *(INSR)* and *KCNJ11* [28]. GWAS among the Europeans have identified T2DM susceptibility loci at *PPARG* (rs1801282) [29]*, KCNJ11* (rs5219) [30], *WFS1* (rs10010131) [31], *IGF2BP2* (rs4402960) [32], *SLC30A8* (rs13266634) [33], *CDKN2A/B* (rs10811661) [33, 34], *HHEX/IDE* (rs1111875) [32], *FTO* (rs8050136) [35], neurogenic locus notch homolog protein 2 (*NOTCH2)* (rs10923931) [36], thyroid adenoma-associated (*THADA)* (rs7578597) [36], *KCNQ1* (rs231362) [36], prospero homeobox protein 1 (*PROX1)* (rs340874) [36], B cell lymphoma/leukaemia 11A (*BCL11A*) (rs243021) [30], glucokinase regulator (*GCKR)* (rs780094) [37], *TCF7L2* (rs7903146) [38]. The Pro12Ala variant of PPARG showed protective effects in Finnish, Czech and Scottish ancestries [39].

#### African regions

Pirie et al. concluded that the risk polymorphisms identified in Caucasian populations were not associated with type 2 diabetes in South African subjects of Zulu descent, except for rs7903146 (*TCF7L2*) [40]. The study analysed rs1801282 (*PPARG*), rs5215 (KCNJ11), rs12255372 (*TCF7L2*), rs7903146 (*TCF7L2*) rs9939609 (*FTO*) and rs1111875 (*HHEX*) which were found to be significant among the European ancestry. At the locus *TCF7L2*, homozygosity for the C allele (CC) was less frequent in the subjects with type 2 diabetes. Heterozygosity (CT) at rs7903146 (*TCF7L2)* occurred more frequently in the subjects with type 2 diabetes. No difference was found between subjects with type 2 diabetes and controls for the TT genotype at rs7903146 [40]. This variant of TCF7L2 was also associated with T2DM in the Western African population [40, 41].

Furthermore, Pirie et al. identified that the Africans have only a homozygous variant of KCJN11, unlike American and European ancestry with heterozygous and homozygous variants. The K variant found significant in European ancestry was rare or non-existent and absent in the Africans [30, 40]. A genome-wide association study of 5000 Africans from Ghana, Nigeria and Kenya identified a novel locus zinc finger RANBP2-type-containing 3 (ZRANB3) gene for T2DM [42]. ZRANB3 is a protein-coding gene with nucleic acid binding and endonuclease activity. The ZRANB3 transcript targets nonsense-mediated decay (NMD) and is expressed in tissues relevant to T2D, including adipose tissue, skeletal muscle, pancreas, and liver [42].

Studies among African Americans showed considerable differences in genetic and non-genetic risk factors (including lifestyle and behavioural factors) with the native African population. African Americans had approximately 20% European admixture [41]. Studies among African Americans showed 30% to 40% higher risk for T2DM among those with the highest tertile of African ancestry [43].

#### American continent

A study by Mercader & Florez, 2017 among the Latino population, solute carrier family 16 Member 11 (*SLC16A11)* (rs77086571), *HNF1A* (rs483353044) and insulin-like growth factor (rs149483638) was found to be significantly associated with T2DM [44]. This variant of insulin-like growth factor was present at approximately 17% in the Mexican population but was rare in European and other populations [44]. The rs483353044 of HNF1A gene was associated with T2DM and was found in 0.36% of individuals without T2D but in 2.1% of participants with the disease [24]. Among the Mexican Americans, ATP-binding cassette transporter (*ABCA1),* adrenoceptor beta 3 (*ADRB3),* calpain 10 *(CAPN10), CDKAL1, CDKN2A/2B,* C-reactive protein *(CRP),* engulfment and cell motility protein 1 *(ELMO1), FTO, HHEX, IGF2BP2,* insulin receptor substrate 1 (*IRS1),* zinc finger protein 1 (*JAZF1), KCNQ1, LOC387761* (a hypothetical gene)*,* lymphotoxin alpha *(LTA),* neurexophilin 1 *(NXPH1),* sirtuin 1 *(SIRT1), SLC30A8, TCF7L2 and* tumour necrosis factor-alpha (*TNF-α)* genes were found to be associated with T2DM [44]. A multi-ethnic study (European Americans, African Americans, Latinos, Hawaiians, Japanese Americans) in America identified rs7578597 of *THADA* as positively associated with European Americans and Native Hawaiians (OR = 1.65, 95% CI = 1.01–2.70) [45]. The rs1801282 of the *PPARG* gene was associated with African Americans. The rs4402960 of *IGF2BP2* was associated with African Americans and Japanese Americans. rs10010131 of wolframin ER transmembrane glycoprotein (*WFS1)* was associated with Latin Americans and Hawaiians. The most commonly studied *TCF7L2 (*rs7903146) was associated with all ethnic groups except the Hawaiians [45].

#### Eastern Mediterranean

A study conducted in Jordan's Circassian and Chechen communities identified two novel SNPs at Jagged canonical Notch ligand 1 (*JAGI)* (rs6134031) and MLX-interacting protein-like (*MLXIP)* (rs4758690) [46]. These two were tested among the Europeans. The SNP, rs6134031 in the Jordan analysis, demonstrated a nominally significant association with T2DM among the Europeans (*P* = 0.012) and the same direction of effect. Serum adiponectin and SNPs in ADIPOQ gene were found to be associated with T2DM in Jordanian population in which the serum adiponectin lowered the risk for prediabetes. At the same time, the GT genotype of rs1501299 increased the risk of prediabetes as well as the TT genotype [47]. A recent study among the Arab population, where consanguineous marriages are more, has identified ribosomal protein S6 kinase B1 (*RPS6KA1)* gene, rs487321 (recessive, intronic, calcium-dependent secretion activator (*CADPS*)), rs707927 (additive, intronic in valyl-tRNA synthetase (*VARS))* and rs12600570 (additive, intronic, DExH-Box Helicase 58 (*DHX58*)). Of these three suggestive markers, the *CADPS* and *VARS* are associated with increased fasting plasma glucose [48]. A systematic review of the Iranian population identified KCNJ11 and TCF7L2 which are strongly associated with T2DM [49].

#### Western Pacific region

A study analysed 14 SNPs at *HHEX, CDKAL1,* cyclin-dependent kinase inhibitor 2B *CDKN2B, SLC30A8, KCNJ11, IGF2BP2, PPARG, TCF7L2, FTO, KCNQ1,* insulin receptor substrate 1 (*IRS1), GCKR,* ubiquitin-conjugating enzyme E2 D2 (*UBE2E2), C2 calcium-dependent domain-containing 4A (C2CD4A/B)* in the Japanese population [50]. Among the 14 SNPs from 14 loci, 4 SNPs (rs7756992 in *CDKAL1*, rs10811661 near *CDKN2B*, rs13266634 in *SLC30A8* and rs2237892 in *KCNQ1*) were found to be significantly associated with T2DM. The association of rs2237892 in *KCNQ1* was the strongest in the Japanese sample, and rs4402960 in *IGF2BP2*, rs2943641 near *IRS1*, rs780094 in *GCKR*, rs7172432 in *C2CD4A*/B and rs5219 in *KCNJ11* showed a positive association with T2DM. In contrast, no association was seen in rs7903146 (*TCF7L2)*, rs1111875 (*HHEX)*, rs1801282 (*PPARG)*, rs8050136 (*FTO)* and rs7612463 (*UBE2E2)* [51], while a genome-wide study among the Australian aboriginals identified association with TCF7L2, potassium inwardly rectifying channel, subfamily J, member 6 (KCNJ6) and melanocortin 4 receptor (MC4R) [52].

## Conclusions

Much of our efforts on diabetes prevention are focused on modifiable behavioural risk factors such as physical inactivity, unhealthy diet and tobacco use. In epidemiological studies, the high-risk population is identified at the community level through risk scores consisting of behavioural risk factors and anthropometric measures such as high body mass index and increased waist circumference. More often, the pathophysiological process would have begun once these risk factors set it. The primarily advocated lifestyle modification for T2DM prevention requires positive reinforcement and a conducive environment for implementation. Although lifestyle modification strategies have been shown to have moderate long-term effects on diabetes prevention, it often requires a favourable non-obesogenic environment for adherence. In this context, understanding the genetic determinants can identify the risk groups prior to the onset of these risk factors.

In this review, we found commonalities in marker–trait associations of specific genes to diabetes (e.g. PPARG, TCFL2) in specific geographical regions. However, it cannot be generalized to all populations as these were found to be population specific.

The SNP rs7903146 of the TCF7L2 gene is the most significant genetic marker associated with type 2 diabetes risk in all the ethnicities. This gene is a transcription factor that influences the transcription of several genes, thereby exerting a large variety of functions within the cell. This might be why the gene is significant in almost all the ethnic groups. PPARG is yet another gene found to be significant in all ethnic groups which regulates fatty acid storage and glucose metabolism. Studies have shown that free fatty acids mediate insulin resistance and impaired glucose tolerance associated with central obesity. PPARG has shown both protective and risk associations with T2DM in several regions. Animal studies have shown that PPARG protects from high-fat diet-induced insulin resistance. A Pro12Ala polymorphism has been detected in humans. This polymorphism might cause a reduction in the transcriptional activity of PPARgamma, leading to decreased insulin resistance and decreased risk of type 2 diabetes. This substantiates that the expression of genes is population specific. FTO gene is associated with obesity, and it has been identified as a risk for the development of T2DM in Indians, Europeans, Africans, Western Pacific and American regions. ABCC8 gene is risky in European as well as Indian populations. Genome-wide association studies have reported that IGF2BP2 disrupts insulin secretion. IGF2BP2 was a risk for T2DM in the Western Pacific, Americas and European ethnicities with no significant role in the Indian population. KCJN11 was associated with T2DM among Western Pacific, Africa and European region but not in Indian population.

India has the second-largest number of people living with diabetes, contributing to high mortality and disability adjusted life years. In our review, we could find evidence of marker–trait associations with type 2 diabetes mellitus from only nine states out of 29 states and seven Union Territories in India. The Indian State of Kerala, despite having the highest prevalence of diabetes in the country, has reported only one study on genetic traits of type 2 diabetes research [15]. This warrants future research on genetic markers of diabetes in India and other regions for developing and identifying biomarkers for screening, prevention and precision medicine.

One of the major limitations we found was that the studies were conducted in non-representative groups within geographical regions, with inherent limitations such as smaller sample sizes or looking into only specific marker–trait associations. Genome-wide association studies using data from large prospective studies are required worldwide to establish the genetic determinants of type 2 diabetes mellitus. This urgently needs to be done in regions with the highest burden of mortality and morbidity related to T2DM. We also need future research to understand genes' special effect and interaction in predicting diabetes mellitus and other comorbidities leading to the highest burden of diseases.

## Data Availability

Not applicable.

## Abbreviations

IDF: International Diabetes Federation
*IRS1*: Insulin Receptor Substrate 1
MENA: Middle East and North African region
MNT: Medical Nutrition Therapy
NCD: Non-Communicable Diseases
OR: Odds Ratio
PMI: Precision Medicine Initiative
*PPARG*: Proliferator-Activated Receptor Gamma
SNP: Single Nucleotide polymorphism
T2DM: Type 2 diabetes mellitus
